# Prospective Study on the Influence of Occupational Hand Protection Products on the Efficacy of Hand Disinfection

**DOI:** 10.3390/healthcare12060646

**Published:** 2024-03-13

**Authors:** Magdalena Metzger, Stefan Manhartseder, Leonie Krausgruber, Carina Wagner, Sara Frank, Rosmarie Reisner, Monika Ehling-Schulz, Johannes Grillari, Roswitha Hosemann, Peter Dungel

**Affiliations:** 1 Ludwig Boltzmann Institute for Traumatology, The Research Center in Cooperation with AUVA, 1200 Vienna, Austria; 2 Austrian Cluster for Tissue Regeneration, 1200 Vienna, Austria; 3 Institute of Molecular Biotechnology, University of Natural Resources and Life Sciences, 1190 Vienna, Austria; 4 Stabsstelle Berufskrankheiten, Abteilung für Berufskrankheiten und Arbeitsmedizin, Allgemeine Unfallversicherungsanstalt AUVA, 1200 Vienna, Austria; 5 Functional Microbiology, Institute of Microbiology, Center of Pathobiology, Department of Biological Sciences and Pathobiology, University of Veterinary Medicine Vienna, 1210 Vienna, Austria; 6 Abteilung Unfallverhütung und Berufskrankheitenbekämpfung, Allgemeine Unfallversicherungsanstalt AUVA, 1100 Vienna, Austria

**Keywords:** hand protection products, barrier creams, skin disinfection, occupational skin diseases, EN 1500

## Abstract

Background: To prevent occupational skin diseases, employees are instructed to periodically apply hand protection products as a barrier to protect their hands from water, cleaning agents or other irritants. The aim of this work was to investigate whether bacteria present on the skin at the time of protection product application are enclosed underneath this protective layer, if they can be transferred to other surfaces and if a standard isopropanol-based skin disinfectant can nonetheless reduce the bacterial burden. Methods: This prospective study was conducted in human volunteers based on the European Standard (EN 1500) to assess the burden of microorganisms before and after the application of various protection product formulations and subsequent hand disinfection. Results: All protection products, with the exception of alcohol-based gels, enclosed bacteria underneath a lipid layer which could be transferred onto other surfaces. Still, the hand disinfectant efficiently reduced the bacteria burden. Discussion: In occupations where proper hand hygiene is vital, alcohol-based gels might be the best option for the protection of the skin barrier as well as for reducing the contamination risk. Conclusion: An alcohol-based disinfection agent can dissolve the lipid film of protection products following the standard protocol for hygienic hand disinfection.

## 1. Introduction

According to the European Agency for Safety and Health at Work (EU-OSHA), dermatologic conditions are among the most prevalent occupational diseases affecting a wide variety of professions, especially hairdressers and cosmetologists, healthcare workers, food handlers, cleaning staff, craftspeople and related professions. The occupational risk factors associated with skin barrier damage are multiple; include wearing gloves, frequent hand washing, cleaning agents or other irritants; and are based on prolonged exposure or contact [1,2,3]. The 2008 European Risk Observatory Report states that around 80% of occupational dermatoses are caused by skin irritations (induced by chemical, biological or physical agents), while approximately 10% result from allergic reactions. The rest originates from other causes such as skin cancers [4]. Occupational skin diseases represent a high socioeconomic burden related to medical expenses and decreased productivity of the employees, who often need to reduce hours, take time off for rehabilitation, change professions or even retire early. Hence, measures must be taken to prevent the development of dermatoses, in particular the use of special skin barrier creams, often termed protection products (PPs). The properties of these PPs have to be matched to the tasks of the given profession. For example, employees exposed to wet work without gloves are advised to use creams with high lipid content due to their tendency to form a protective lipid film. On the contrary, when using gloves, lipid-containing creams would produce an overly occlusive effect. Besides skin PPs, in many occupational fields, the use of hand disinfection products is necessary as well. Especially in the healthcare sector as well as in the food industry, employees have to use antiseptics numerous times per day as clinically relevant pathogens can be easily transferred by human touch. Pittet et al. showed the potential risk of transfection during routine patient care such as manual blood pressure measurements from a patient’s wrist [5,6]. The frequent use of disinfectants (mostly alcohol solutions) has been associated with a disruption of the skin barrier. Therefore, to prevent this adverse reaction, skin protectants are recommended and often mandatory.

Despite the frequent and necessary use of both agents, there are limited data on the effects of hygienic hand disinfectants after the use of occupational skin protection agents. The question of whether the protectant application masks the bacteria present on the skin and thus lowers the effectiveness of subsequent hand disinfection thus arises. Therefore, the risk of cross-contamination could be increased, relevant for healthcare and food industry workers.

The aim of the present study was to investigate the effects of skin protection agents on artificially contaminated skin in human volunteers and the success of subsequent hand disinfection.

## 2. Materials and Methods

The examination procedure was based on the EN 1500 standard and was modified to test the effects of skin PPs. Eight different hand PPs were tested either alone or with subsequent hand disinfection. Three different treatment groups were examined: (1) hand disinfection, (2) hand PPs, and (3) first PP application and subsequent disinfection.

Desmanol^®^ pure (Schülke & Mayr GmbH, Norderstedt, Germany), an alcohol-based skin disinfection product containing 75 g of propan-2-ol in a 100 g solution was used.

Hand PPs were grouped into one of three categories based on their galenic formulations:

(1) Alcoholic gels:
  A: Pevasan Gel, Paul Voormann GmbH, Velbert, Germany;
  B: Proglove Gel, Physioderm, Euskirchen, Germany.
(2) Water-insoluble creams (water-in-oil emulsions):
  C: Protexan^®^, Physioderm, Euskirchen, Germany;
  D: Pevasan SF, Paul Voormann GmbH, Velbert, Germany;
  E: Saniwip^®^, Physiodem, Euskirchen, Germany;
  F: Softhandcreme, Allergika Pharma GmbH, Wolfratshausen, Germany.
(3) Water-soluble creams (oil-in-water emulsions):
  G: Pevaperm, Paul Voormann GmbH, Velbert, Germany;
  H: Stokoderm^®^ Protect Pure, SC Johnson Professional USA, Inc., Racine, WI, USA.

A more detailed list with the ingredients of the products can be found in the Supplementary Materials, in Tables S1 and S2.

### 2.1. Study Design

This study was conducted at the Ludwig Boltzmann Institute for Traumatology in accordance with the Declaration of Helsinki and approved by the Ethics committee for AUVA hospitals, Austria, 11/2021. Thirty healthy volunteers (including co-workers) with no skin diseases and no visible skin lesions were chosen to participate in this study and were subjected to the experimental procedure at least three days apart. Informed consent was obtained from all subjects prior to participation. 

**Figure 1:**
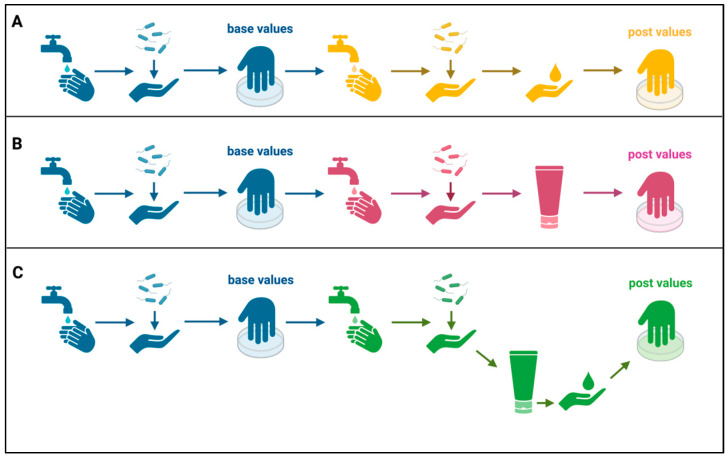
Schematic overview of the experimental design: First, participants washed their hands, which were contaminated with a non-pathogenic bacterial strain. Bacterial burden was determined by immersing the fingertips into PBS and massaging the bottom of a Petri dish (base values). After washing again and re-contamination, either skin disinfectant (**A**), one of eight hand protection products (**B**) or a combination of both (**C**) were applied before the bacterial count (post values) was determined again. Image created with Biorender.com.

After participants washed their hands with medical soap (Baktolin^®^ pure, Paul Hartmann AG, Heidenheim an der Benz, Germany) for 1 min and dried their hands with a paper towel, 3 mL of bacterial suspension containing 2 × 10^8^ colony forming units per mL (CFU/mL) were pipetted into the palm of one hand. Hands were then rubbed together for 30 s following the standard hand-rubbing procedure explained in EN 1500. After air-drying for 2 min, the base values of bacterial contamination were taken by immersing the fingertips and massaging the bottom of two Petri dishes filled with 10 mL of phosphate-buffered saline (PBS, Lonza, Basel, Switzerland) for 1 min. Participants then washed their hands again before they were re-contaminated as described above. After the second 2 min air-drying step, depending on the respective experimental group, either solely a hand disinfectant, a PP or the combination of both was applied:

(1) Application of PPs: Appropriate amounts of PPs (see Supplementary Table S3) were distributed onto both hands of the participants for 1 min following the standardized rubbing procedure. To let the PPs soak in, hands were left to dry for 1 min. In the respective groups, the hand disinfectant was applied immediately after.
(2) Application of skin disinfectant: Either directly after re-contamination (disinfection only group) or after PP application and air-drying, 3 mL of the antiseptic was pipetted into the palm of the hand, which was then rubbed in for 30 s according to the test procedure EN 1500.
(3) Directly afterwards, post values were taken to quantify the bacterial burden in the same way as the base values. See Figure 1 for a schematic overview of the experimental procedures.

### 2.2. Preparation of the Contamination Fluid 

All chemicals were purchased from Sigma Aldrich, St. Louis, MO, USA, unless stated otherwise. The bacterial strain predefined in EN 1500 is *Escherichia coli* K12 (*E. coli* K12; Addgene, Watertown, NY, USA), which was grown overnight in tryptic soy broth (TSB) at 37 °C in a shaking incubator at 250 rpm (Edmund Bühler GmbH, Bodelshausen, Germany). The optical density at 600 nm (OD_600_) was determined via a spectrophotometer (Hitachi High-Technologies Corporation, Tokio, Japan), and cell count was calculated using the relation OD_600_ *E. coli* K12 = 1 $\hat{=}$ 4 × 10^8^ CFU/mL. The bacterial suspension was adjusted to a concentration of 2 × 10^8^ CFU/mL with PBS. It was used within three hours of preparation.

### 2.3. Quantification of Bacterial Burden (Processing of Base and Post Values)

In order to determine the bacterial burden before and after using a test product, participants immersed the fingertips of both their hands separately in a Petri dish filled with 10 mL of PBS. Within 30 min, a 1 mL sample of each Petri dish was transferred into microcentrifuge tubes and serial dilutions were prepared in PBS. Several 20 µL drops of each dilution were pipetted onto tryptone soy agar (TSA) plates and grown overnight at 37 °C (Thermo Electron LED GmbH, Langenselbold, Germany). On the next day, distinct colonies could be counted, and the mean bacterial concentration of six technical replicates was calculated.

### 2.4. Determining Antibacterial Properties of the Hand Protection Products Used

To test the antibacterial properties of hand PPs, 100 µL of the *E. coli* K12 overnight suspension was plated onto TSA plates and a 10 µL drop of each PP was added on top using a positive displacement pipette. After overnight incubation, growth patterns around the PPs were observed.

### 2.5. Swabbing Procedure and Transfer Tests 

It was hypothesized that bacteria were enclosed underneath the lipid film of PPs, and their presence could not be quantitatively determined using the standard liquid sampling technique described above. Therefore, swabs were taken as a qualitative control method. Sterile swabs (Nobamed Paul Danz AG, Wetter, Germany) were pre-saturated with PBS before swabbing a defined, approximately 2 cm^2^ sized region on one palm of the hand for 30 s. Then, the swab was put into a microcentrifuge tube filled with 500 µL PBS and stirred for 1 min. Next, 100 µL thereof was transferred onto a TSA plate and spread with a sterile spreader rod. The plates were incubated at 37 °C and checked for the presence of bacterial colonies on the next day. Furthermore, to test the transfer of entrapped bacteria onto other surfaces, imprints of the participants’ thumbs were taken: imprints of the left thumb were taken after washing, re-contamination with *E. coli* K12 and application of a PP, whereas imprints of the right thumbs were taken after re-contamination and therefore served as controls.

### 2.6. Statistical Analysis

Experimental results were tested for significance using ordinary one-way ANOVA with Dunn’s multiple comparisons test comparing all treatment groups to base values in GraphPad Prism 9 (GraphPad Software, Boston, MA, USA). Statistical significance was accepted for a *p*-value ≤ 0.05.

## 3. Results

### 3.1. Antibacterial Effects of the Chosen Hand Protection Products

**Figure 2:**
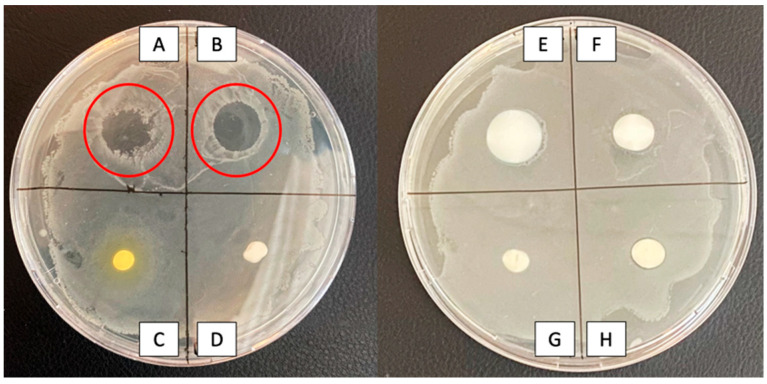
Antimicrobial effects of the various hand protection products: only products (**A**,**B**) (alcoholic gels) demonstrated bactericidal action by inhibiting the growth of *E. coli* K12. Neither water-insoluble creams (**C**–**F**) nor water-soluble ones (**G**,**H**) had any effect.

To test whether the hand PPs have antimicrobial properties on their own, *E. coli* K12 was plated onto TSA plates and the products were added on top. There was a clear growth-inhibiting effect of the alcoholic gels (products A and B), indicated with a red circle in Figure 2, while all other products did not affect bacterial growth.

### 3.2. All Non-Alcoholic Skin Protection Enclosed E. coli K12 Underneath a Lipid Film

**Figure 3:**
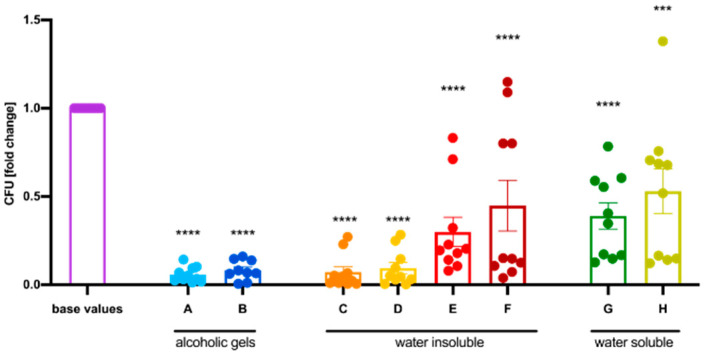
Colony forming units (CFUs) were significantly decreased after the use of all tested hand protection products (A–H) compared to the CFUs before (base values). The test subjects’ fingertips were immersed into the collection fluid, and bacteria that were washed into the solution were quantified. Low numbers of CFUs indicated either antibacterial effects of the hand protection product or entrapment of the bacterial strain underneath a lipid layer. There is a trend towards a higher amount of CFUs detected after products that contained a larger number of water-soluble ingredients were used. Mean ± SEM, one-way ANOVA with Dunnett’s multiple comparison test, *n* = 5 participants (10 hands), *** *p ≤* 0.001, **** *p ≤* 0.0001.

**Figure 4:**
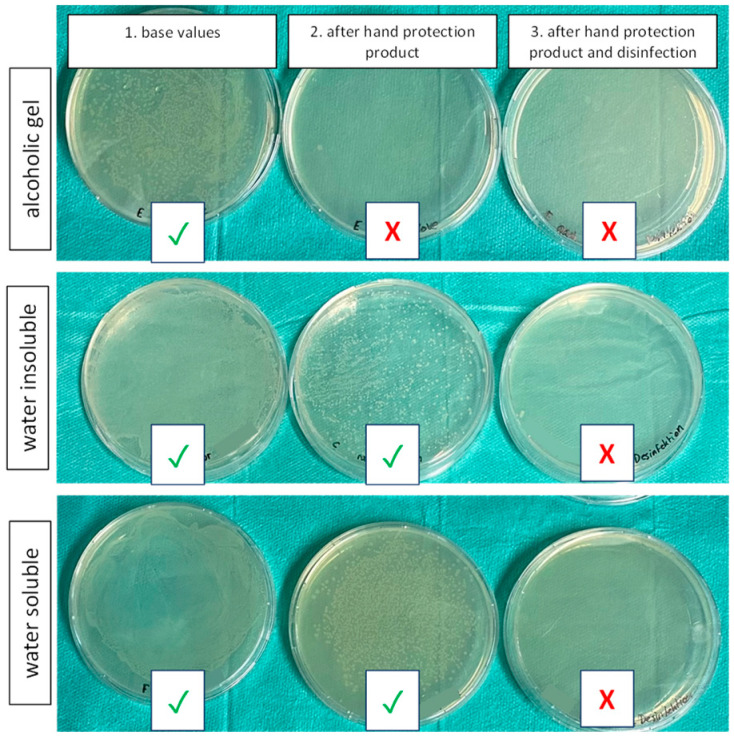
Samples taken via sterile swabs after contamination before the use of any product (1), after the hand protection product was dispersed (2) and after the subsequent use of the hand disinfection product (3). A green checkmark indicates the growth of colonies, while a red cross marks the absence of them. While the alcoholic gels exerted a strong antibacterial action, we detected a substantial number of colonies in the water-insoluble and water-soluble groups. Still, the application of the hand disinfectant diminished bacterial growth in all three categories. One representative product was chosen here for each group, and detailed pictures of all products are supplied in the Supplementary Materials.

Determined via the described method, all products significantly reduced the number of bacteria compared to values taken without any disinfection or PPs (baseline values). Alcohol-containing gels (Figure 3A,B) resulted in the most pronounced reduction in CFUs, due to their inherent antibacterial action shown above. In addition, all other emulsion-based products (Figure 3C–H) also produced significant declines in CFUs, which posed the question of whether bacterial cells were entrapped underneath the lipid film. Standardized swabs of the test subjects’ hands after applying the respective products showed the presence of bacteria in all groups except the alcohol-containing gels (representative pictures of each category can be seen in Figure 4, and pictures of all PPs can be found in the Supplementary Materials Figures S1–S3), suggesting that microorganisms were trapped underneath a lipid film. Detailed reduction values can be found in Table 1.

### 3.3. Entrapped Bacteria Are Transferrable onto Surfaces 

**Figure 5:**
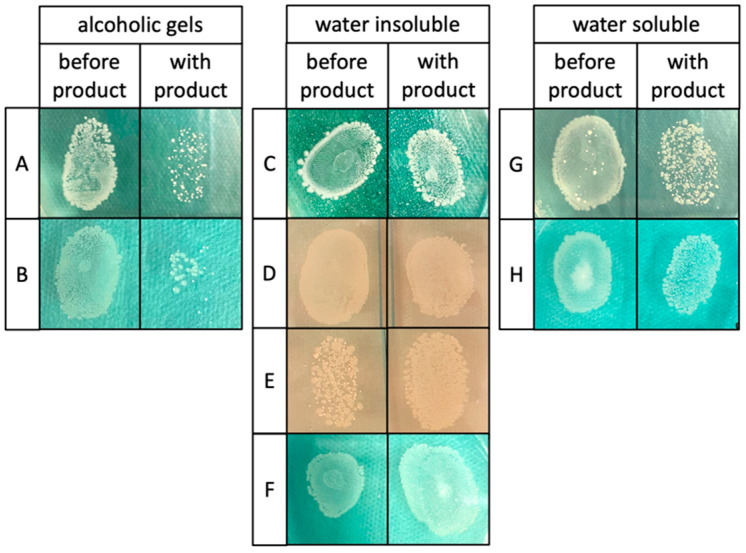
Fingerprints before and after the use of hand protection products. *E. coli* K12 was detected after the use of water-insoluble (**C**–**F**) as well as water-soluble (**G**,**H**) products, indicating that contamination can be transferred to other objects or surfaces. Although alcoholic gels (**A**,**B**) had a reducing effect, still, bacteria were detectable after use of the respective product.

The transfer of bacteria was checked by taking thumbprints after contamination of the hands and after the use of a hand PP. A large number of colonies grew on the agar plates after using PPs from the water-soluble and water-insoluble groups, suggesting that entrapment of bacteria underneath a lipid layer did not prevent them from being transferred to other surfaces (Figure 5). Since the alcoholic gels exerted antibacterial properties (see Figure 2), these products led to a significantly reduced amount of *E. coli* K12 colonies.

### 3.4. Disinfection Was Effective despite Using a Hand Protection Product Beforehand

**Figure 6:**
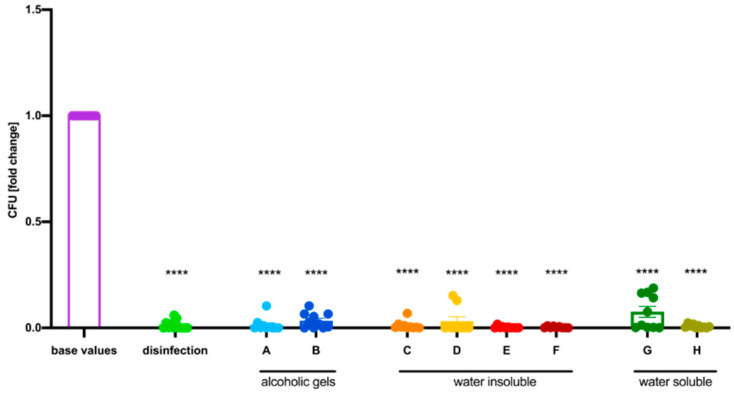
Disinfecting hands after using the respective hand protection products (A–H) reduced the colony forming units (CFUs) of *E. coli* K12 comparable to a group that used only the hand disinfectant (termed disinfection). The displayed values are normalized to the base values, which are the mean number of colonies determined after contaminating the hands of participants with a bacterial suspension containing 2 × 10^8^ CFU/mL. Mean ± SEM, one-way ANOVA with Dunnett’s multiple comparison test, *n* = 5 participants (10 hands), **** *p* ≤ 0.0001.

One major aim of this study was to test if the use of hand disinfectant reduces the bacterial load of *E. coli* K12 despite prior use of hand PPs. While the use of the isopropanol-based disinfectant alone caused a mean decrease of 98.62%, the combination of all investigated hand PPs together with subsequent hand disinfection resulted in a similar reduction in CFUs (Figure 6). Detailed numbers can be found in Table 1. The results were verified with standardized swabs (see Figure 4).

## 4. Discussion

Occupational skin diseases represent a major challenge to healthcare and socioeconomic costs. In many countries, hand PPs are part of personal protective equipment (PPE) and employers are required by law to provide them and to teach proper use. The current recommendation by the Austrian Workers’ Compensation Board (AUVA) is to use an appropriate PP at the beginning of a work shift and a couple times in between to protect the skin from irritations and allergic reactions. After work, employees are encouraged to use hand lotion to enhance skin regeneration. While manufacturers often distinguish between skin protection and skin care products, these terms are not clearly defined and there is no specific characterization of these products. Hence, it can be difficult to choose an appropriate product for particular occupations or tasks. For instance, Gina et al., 2023, tested seven different PPs that were marketed to be used before wearing gloves in a single-blinded randomized controlled study. For seven consecutive days, the PPs were applied before a glove occlusion period. Interestingly, PPs did not mitigate the skin’s susceptibility to the model detergent sodium dodecyl sulfate (SDS). After an irritation challenge, some even aggravated irritation compared to occlusion alone [7]. Other studies have shown that occlusion can decrease the skin’s barrier function after prolonged wear, which may cause irritation and worsening of pre-existing skin diseases [8,9,10].

Skin disinfectants are also part of the PPE in some occupations for preventing the spread of germs and infectious diseases. Pre-existing skin diseases were shown to worsen following the cumulative application of n-propanol, a common ingredient in skin disinfectants, highlighting the importance of proper skin care and protection [10]. 

To date, no standardized testing method for investigating the influence of hand lotions or barrier creams has been established. Therefore, we adapted the EN 1500 standard for testing chemical disinfectants and antiseptics to fit our requirements. According to the EN 1500 standard, all participants have to immerse their hands into the same batch of contamination fluid. In order to avoid possible cross-contamination of other bacterial strains and to always have a homogenous suspension of *E. coli* K12, we decided to store the contamination fluid in a sterile 50 mL tube and distribute it using a sterile pipette. 

Still, in some cases, a specific bacterial strain was detected among the *E. coli* K12 colonies. By matrix-assisted-laser-desorption/ionization time-of-flight (MALDI TOF) mass spectrometry (as previously described by Ballas et al. [11] and in the Supplementary Materials), the strain was identified as *Staphylococcus warneri*, a frequently found commensal of the human skin flora [12]. Due to its morphology, it was easily distinguishable from *E. coli* K12. Therefore, we decided to include experimental runs where a negligible number of *S. warneri* colonies were present.

Furthermore, the EN 1500 standard suggests taking base values, then applying the hand disinfectant, and then immediately taking another sample (post values). Interestingly, when replicating this protocol, we found that even without the use of an antiseptic, post values were decreased by a mean of 80.73% (*n* = 3, Supplementary Materials, Figure S4). This indicated that during the first sampling step, a certain number of bacteria were washed away, automatically decreasing the post values taken right after. Therefore, we implemented a second washing and re-contamination step. 

We demonstrated that most PPs did enclose the used bacterial strain underneath a lipid film since they were not detectable using a liquid sampling method. The effect was more pronounced in water-insoluble than water-soluble products. However, by using a standardized swabbing method to scrape off the lipid layer, we revealed that the bacterial strain was still present and could be transferred to a culture plate. In the food or healthcare industries, this could entail cross-contamination of produce, or between employees and/or patients. Nonetheless, the alcohol-based hand disinfectant was still able to penetrate through the lipid layer formed by the PPs and inactivated the bacterial cells, as shown via the liquid sampling and swabbing methods. In contrast to the water-insoluble and water-soluble PPs, alcoholic gels alone were able to decrease the bacterial contamination already by an average of 94.22% (product A) and 91.83% (product B). Adding the hand antiseptic 3 min afterwards further reduced the CFUs by an average of 98.53% and 96.59%, respectively. 

This is in line with the data from Paula et al., 2017, who did not observe an adverse effect on the efficacy of an alcohol-based hand antiseptic after the use of a hand lotion; however, they tested only one product, which was a water-in-oil emulsion [13]. 

The present study highlights the importance of choosing appropriate detection methods since the liquid samples taken omitted the presence of bacteria trapped underneath a lipid film. Additionally, it is not clear if and how long entrapped bacteria would be able to survive. It can be expected that facultative anaerobic bacterial strains can endure this low-oxygen environment for some time; however, this needs to be addressed in future investigations. It would also be of high interest to repeat the protocol using different bacterial strains such as Gram-positive ones to test possible differences in disinfection efficacy. Further limitations of the present study include the fact that no statement can be made regarding the effects of the applied protocols on skin integrity and barrier function. These aspects need to be addressed in future studies. 

Considering these results, for some occupations, alcohol-based gels might be the preferred choice of PP to prevent the spread of germs, though it should be noted that the reduction in bacterial burden was slightly less effective when using the alcohol-based gels alone, compared to the standalone use of hand disinfectant. Additionally, these gels are listed for application when wearing gloves, routine requirement in the mentioned occupational fields. However, a more in-depth understanding of the effects of multiple applications of both agents on the skin’s integrity needs to be the assessed in additional future studies.

## 5. Conclusions

In summary, occupational hand PPs are an important tool to protect the hands from chemicals, irritants and wet work, among other things. Furthermore, the use of skin disinfectants is also needed in certain fields of work. We observed—with the exception of alcohol-based gels—that bacterial cells can be trapped underneath a lipid film of water-soluble and -insoluble creams which can be transferred to other surfaces. The tested alcohol-based skin disinfectant agent dissolved the lipid film and efficiently reduced the bacterial contamination of hands.

## Figures and Tables

**Table 1:** Mean values of reduction in colony forming units in percent using the liquid sampling method of EN 1500. High numbers of bacterial reduction without disinfection suggested that the presence of a lipid film entrapped bacteria underneath and underlined the need for a second test method.

		Reduction without Disinfection [%]	Reduction with Disinfection [%]
Disinfection product only		-	98.62
Alcoholic gels	A	94.22	98.53
	B	91.83	96.59
Water insoluble	C	92.81	98.85
	D	90.63	96.80
	E	70.03	99.67
	F	55.21	99.67
Water soluble	G	61.06	92.41
	H	47.04	99.22

## Data Availability

The data presented in this study are available from the corresponding author on request.

## Supplementary Materials

The following supporting information can be downloaded at https://www.mdpi.com/article/10.3390/healthcare12060646/s1, Figure S1: Swabs of alcoholic gels; Figure S2: Swabs of water-insoluble protection products; Figure S3: Swabs of water-soluble protection products; Figure S4: The liquid sampling step of the DIN EN 1500 protocol reduced the number of bacteria present on the hands; Table S1: List of ingredients; Table S2: Ingredient list of the hand wash and hand disinfection products used; Table S3: Amount of protection products applied to hands depending on the glove sizes of the participants.
