# Supplementary material for: Prospective Study on the Influence of Occupational Hand Protection Products on the Efficacy of Hand Disinfection
Source: Healthcare (Basel). 2024 Mar 13;12(6):646. doi: 10.3390/healthcare12060646 (PMC10969782; doi:10.3390/healthcare12060646)
Supplement: Supplementary file 1 [file healthcare-12-00646-s001.zip › supplements/Supplemental material_hand disinfection study.dotx]

**Supplemental Material**

1. Lists of ingredients

Table S1: Ingredient list of the hand protection products used

|  | | **Name** | **Manufacturer** | **Ingredients** |
| --- | --- | --- | --- | --- |
| **Alcoholic Gels** | **A** | Pevasan Gel | Paul Voormann GmbH, Velbert, Germany | Alcohol Denat., Aqua, Isopropyl Alcohol, Hamamelis Virginiana Leaf Water, Glycerin, Panthenol, Bisabolol, Propylene Glycol, Acrylates/C10-30 Alkyl Acrylate Crosspolymer, Aminomethyl Propanol |
|  | **B** | Proglove Gel | Physioderm, Euskirchen, Germany | Alcohol Denat., Aqua, Propylene Glycol, Isopropyl Alcohol, Hamamelis Virginiana Leaf Water, Glycerin, Acrylates/C10-30 Alkyl Acrylate Crosspolymer, Tetrahydropropyl Ethylenediamine, Carboxymethyl Chitin, Bisabolol |
| **Water insoluble Creams (Water-in-Oil Emulsions)** | **C** | Protexan® | Physioderm, Euskirchen, Germany | Aqua, Caprylic/Capric Triglyceride, Glycerin, Butyrospermum Parkii Butter, Behenyl Alcohol, Alcohol Denat., Squalane, Oryza Sativa Bran Cera, C12-16 Alcohols, Hydrogenated Lecithin, Palmitic acid, Reseda Luteola Extract, Tocopherol, Ubiquinone, Ceramide NP, VP/Eicosene Copolymer, Glyceryl Caprylate, Nylon-6, Acrylates/Vinyl Isodecanoate Crosspolymer, Dehydroxanthan Gum, Sodium Carbomer, Sodium Anisate, Sodium Levulinate, Citric acid |
|  | **D** | Pevasan SF | Paul Voormann GmbH, Velbert, Germany | Aqua, Paraffinum Liquidum, Caprylic/Capric Triglyceride, Isostearyl Diglyceryl Succinate, Paraffin, Stearalkonium Hectorite, Propylene Carbonate, Parfum, Benzyl Alcohol, PPG-2 Methyl Ether, 2-Bromo-2-Nitropropane-1,3-Diol, Deceth-8, Iodopropynyl Butylcarbamate |
|  | **E** | Saniwip® | Physioderm, Euskirchen, Germany | Aqua, Paraffinum Liquidum, PEG-7 Hydrogenated Castor Oil, Isopropyl Myristate, Petrolatum, Sorbitol, PEG-45/Dodecyl Glycol Copolymer, Lanolin, Phenoxyethanol, Sorbit Isostearate, PEG-2 Hydrogenated Castor Oil, Magnesium Sulfate, Ethylhexylglycerin, Sorbic acid, Lecithin, Propylene Glycol, BHT, Glyceryl Stearate, Ascorbyl Palmitate, Citric acid |
|  | **F** | Softhandcreme | Allergika Pharma GmbH, Wolfratshausen, Germany | Aqua, Paraffinum Liquidum, Isopropyl Myristate, Glycerin, Cetyl PEG/PPG-10/1 Dimethicone, Sodium Chloride, Polyglyceryl-4 Isostearate |
| **Water soluble Creams (Oil-in-Water Emulsions)** | **G** | Pevaperm | Paul Voormann GmbH, Velbert, Germany | Aqua, Glyceryl Stearate, Sorbitol, Myristyl Lactate, Triacontanyl PVP, Ceteareth-20, Cetearyl Alcohol, Ceteareth-12, Cetyl Palmitate, Guar Hydroxypropyltrimonium Chloride, Bisabolol, Parfum, Sodium Diethylenetriamine Pentamethylene Phosphonate, Benzyl Alcohol, Benzoic Acid, Dehydroacetic Acid |
|  | **H** | Stokoderm® Protect Pure | SC Johnson Professional USA, Inc., Wisconsin, United States | Aqua, Kaolin, Cetearyl Alcohol, Dicapryly Carbonate, Glycerin, Xanthan Gum, Phenoxyethanol, Diazolidinyl Urea, Sodium Lauryl Sulfate, Sodium Cetearyl Sulfate, CI 15985, CI 19140. |

Table S2: Ingredient list of the hand wash and hand disinfection product used

|  | **Name** | **Manufacturer** | **Ingredients** |
| --- | --- | --- | --- |
| Hand Wash | Baktolin® pure | Paul Hartmann AG, Heidenheim an der Benz, Germany | Aqua, Sodium Lauryl Sulfate, Sodium Chloride, PEG-7 Glyceryl Cocoate, Cocamidopropyl Betaine, Glycerin, Disodium Lauryl Sulfosuccinate, Sodium Benzoate, PEG-120 Methyl Glucose Dioleate, Sodium Citrate. |
| Hand Disinfectant | Desmanol® pure | Schülke & Mayr GmbH, Norderstedt, Germany | Tetradecanol, 75 g propan-2-ol in 100 g solution |

1. The amount of protection products (PP) required to be evenly spread on the hands was tested with a fluorescent hand cream to see their distribution, and then tailored to the size of gloves usually worn by the participants:

Table S3: Amount of protection products applied to hands depending on the glove sizes of the participants.

| Glove size | Amount of Protection Products |
| --- | --- |
| S | 600 mg |
| M | 700 mg |
| L | 800 mg |
| XL | 900 mg |

1. Pictures of all swabs


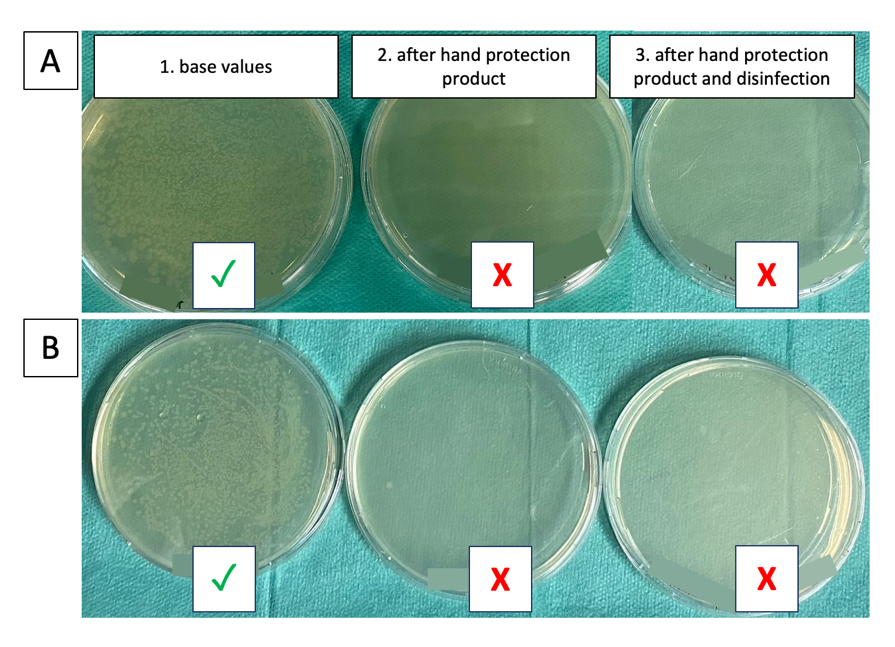


Figure S1: **Swabs of alcoholic gels (A + B):** Participants distributed the bacterial suspension on their hands and after two minutes of air drying, swabs were taken to assess the presence of E. coli K12 (first plate, **base values**). Next, the hand protection product was rubbed into the hands and swabs were taken again from a different site (second plate, **after hand protection product**). Lastly, the hand disinfection product was applied before taking swabs one more time (third plate, **after hand protection product and disinfection**). Green checkmarks indicate a clear presence of E. coli K12, while a red X marks the absence.


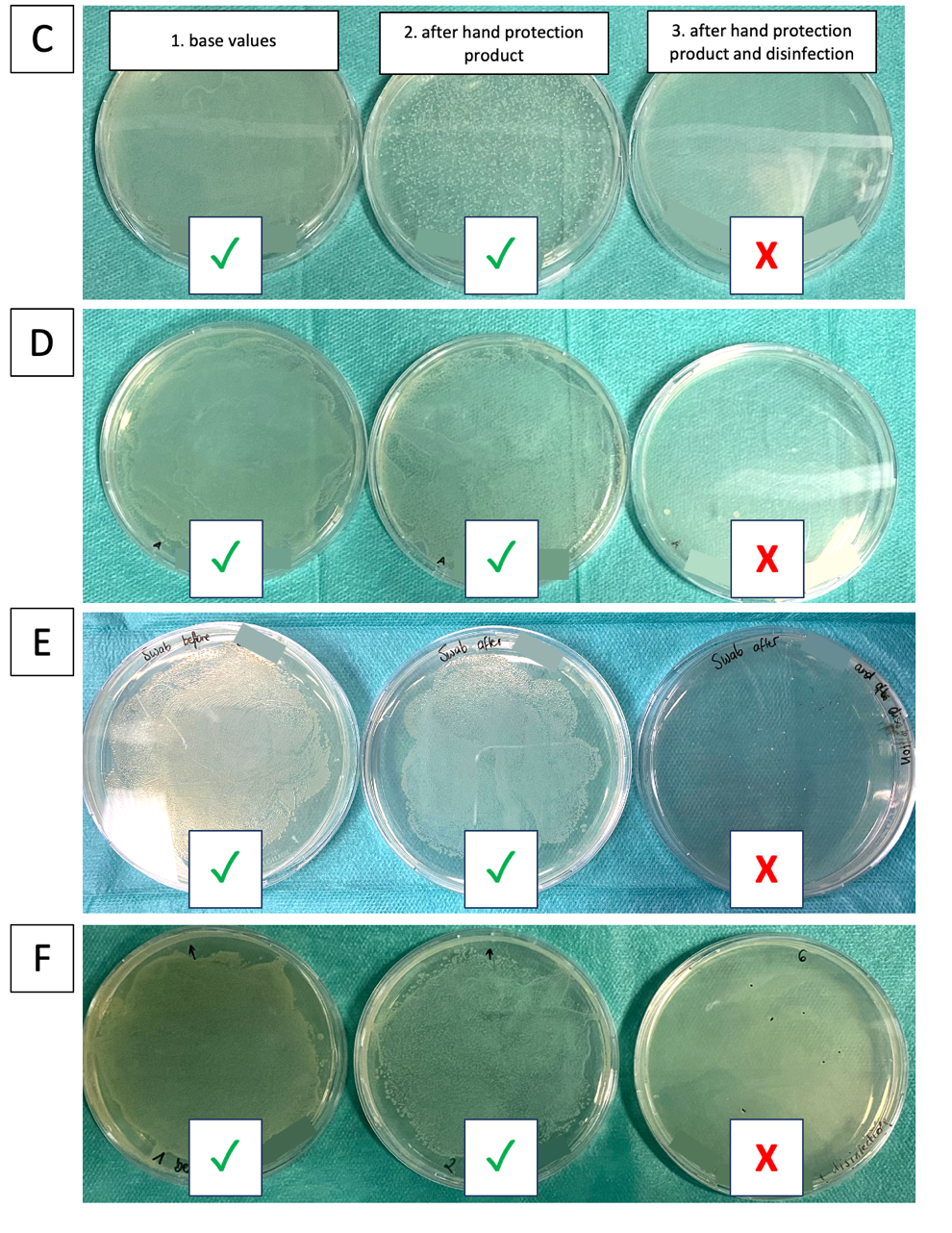


Figure S2: **Swabs of water insoluble protection products (C - F):** Participants distributed the bacterial suspension on their hands and after two minutes of air drying, swabs were taken to assess the presence of E. coli K12 (first plate, **base values**). Next, the hand protection product was rubbed into the hands and swabs were taken again from a different site (second plate, **after hand protection product**). Lastly, the hand disinfection product was applied before taking swabs one more time (third plate, **after hand protection product and disinfection**). Green checkmarks indicate a clear presence of E. coli K12, while a red X marks the absence.


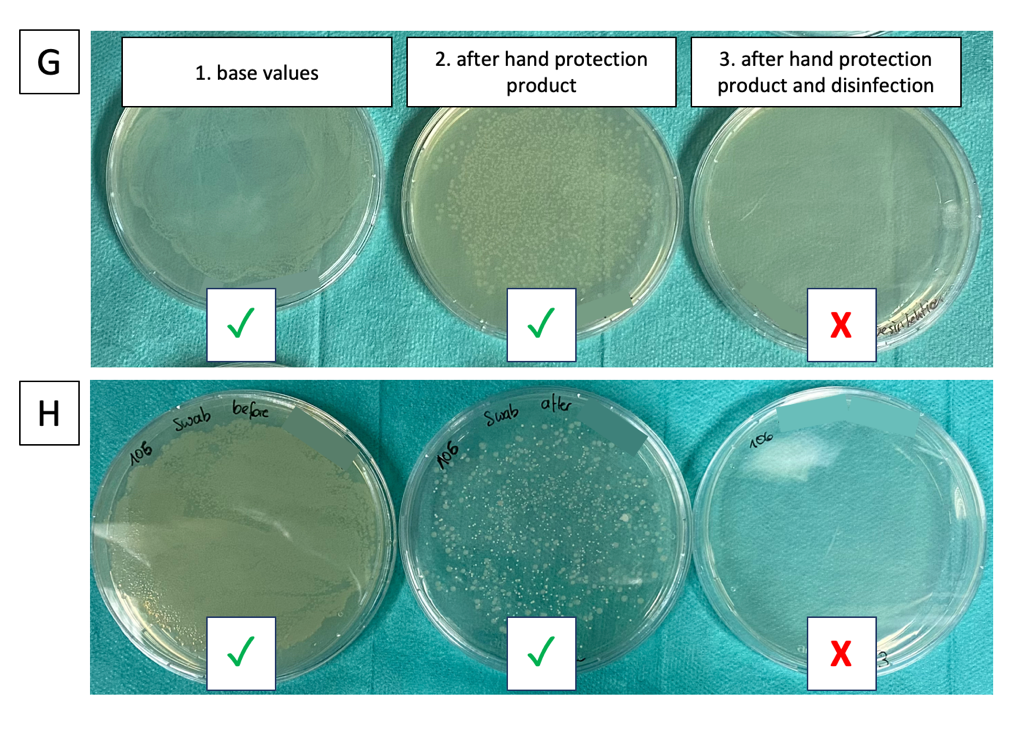


Figure S3: **Swabs of water soluble protection products (G+ H):** Participants distributed the bacterial suspension on their hands and after two minutes of air drying, swabs were taken to assess the presence of E. coli K12 (first plate, **base values**). Next, the hand protection product was rubbed into the hands and swabs were taken again from a different site (second plate, **after hand protection product**). Lastly, the hand disinfection product was applied before taking swabs one more time (third plate, **after hand protection product and disinfection**). Green checkmarks indicate a clear presence of E. coli K12, while a red X marks the absence.

1. Necessity of recontamination:

The DIN EN 1500 standard, which describes a protocol for the testing of hygienic hand disinfection products, requires participants to contaminate their hands with a bacterial test strain before taking samples by inserting the fingertips into phosphate buffered saline (PBS). Next, participants would receive the disinfection product, rub it in and again immerse the fingers in fresh PBS. When participants were instructed to rub their hands together after the sampling step without any disinfection product, bacteria numbers were reduced by 80.73 % (mean value, n = 3) in the second sampling step compared to before. This indicates that the liquid sampling washes away a significant amount of the bacterial cells. Therefore, any subsequently applied test product would start off with a lower bacterial burden and might distort results. Hence, we included a second washing and recontamination step in the setup.


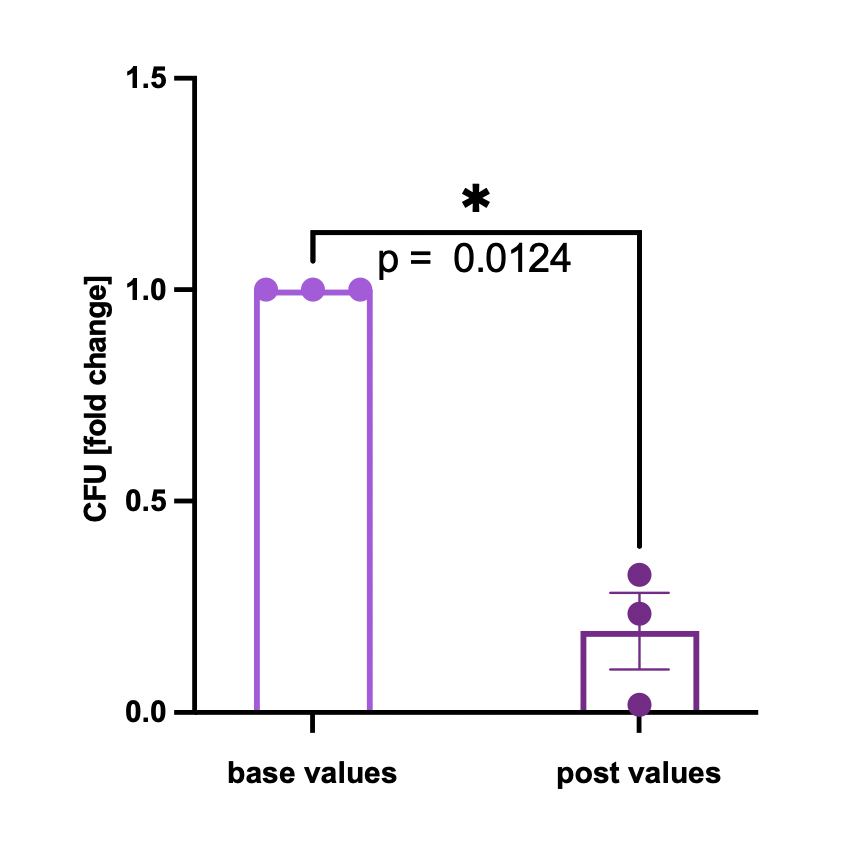


**Figure S4: The liquid sampling step of the DIN EN 1500 protocol reduced the number of bacteria present on the hands**. Participants distributed E. coli K12 on their hands by rubbing them together for 30 sec. After air drying, the fingertips were immersed in PBS for 1 min and colony forming units (CFU) were determined via microbiological methods (**base values**). Right after the liquid sampling step, participants rubbed their hands together using the remaining PBS on their fingertips as lubricant. Next, bacteria numbers were determined again (**post values**). Post values were decreased by 80.73 % compared to base values. (n = 3 participants (6 hands), Paired t-test, Mean ± SEM, * p ≤ 0.05)

1. Identification of the bacterial strain by matrix assisted laser desorption/ionization-time of flight mass spectrometry (MALDI-TOF MS)

Bacterial colonies were isolated from TSA plates and streaked onto a fresh plate for overnight incubation at 37°C. Cells were transferred to a stainless steel 96-well plate (Bruker Daltonics, Bremen, Germany). After drying at room temperature, 1 µl of matrix solution (α-hydroxycinnamic acid, HCCA, Bruker Daltonics, Bremen, Germany) was added on top. Samples were dried again and subsequently measured in the MALDI-TOF MS Microflex device (Bruker Daltonics, Bremen, Germany). Measurements were performed according to the manufacturer’s instructions. For the spectral analyses, the MBT Compass Explorer software (ver. 4.1.60, Bruker, server database: 4.1.60 (PYTH) 28 2016-04-18_11-26-19; Bruker) was used. Score values below 1.70 were not accepted and regarded as unreliable. Scores > 2.0 were regarded as species level identification.

The bacterial strain frequently found on plates of the present study was thereby identified as *Staphylococcus warneri*.
