# Supplementary figures and images for: Prospective Study on the Influence of Occupational Hand Protection Products on the Efficacy of Hand Disinfection
Source: Healthcare (Basel). 2024 Mar 13;12(6):646. doi: 10.3390/healthcare12060646 (PMC10969782; doi:10.3390/healthcare12060646)

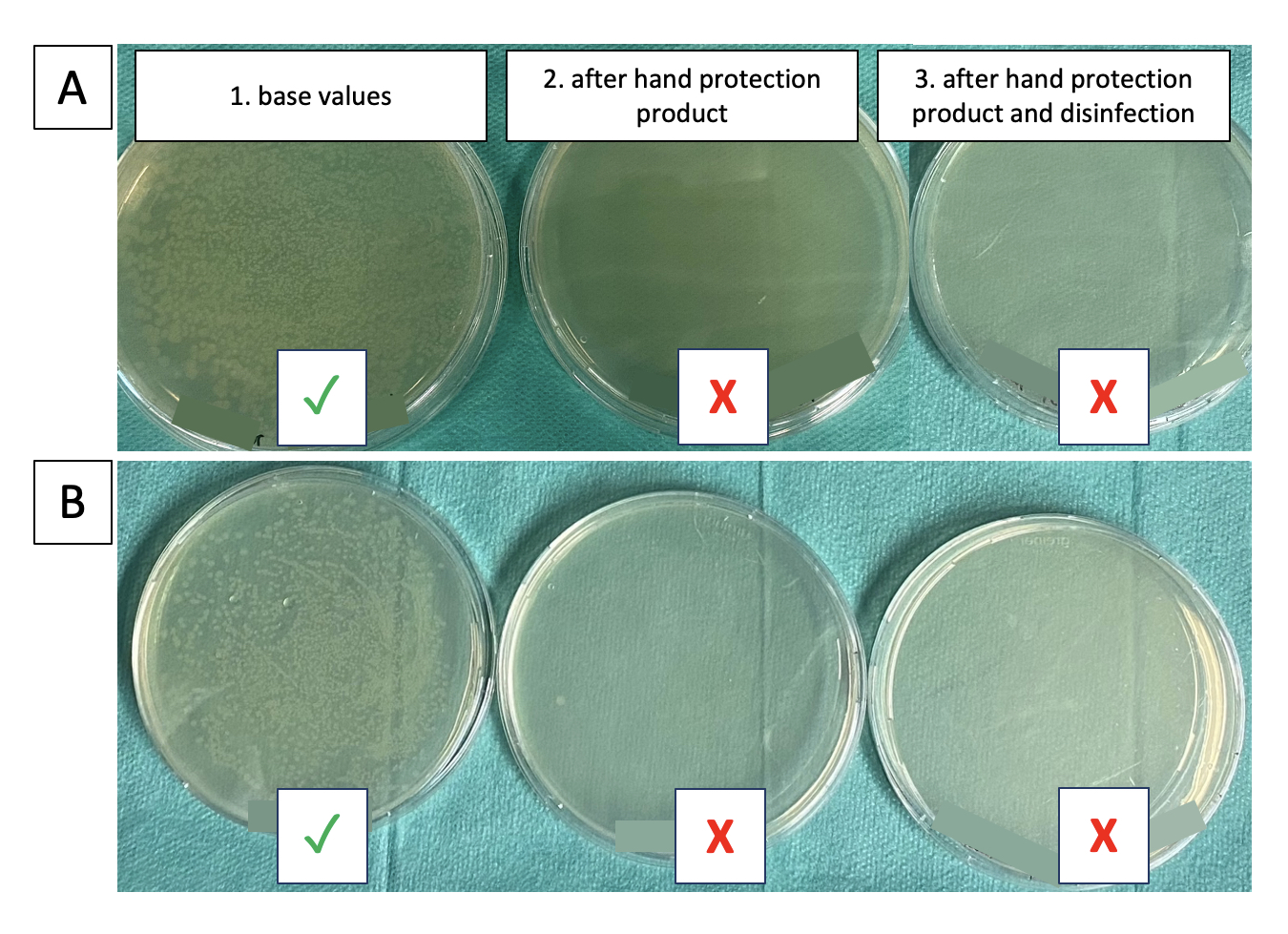

Supplement: Supplementary file 1 [file healthcare-12-00646-s001.zip › supplements/figure S1_all swabs_alcoholic gels.jpg]

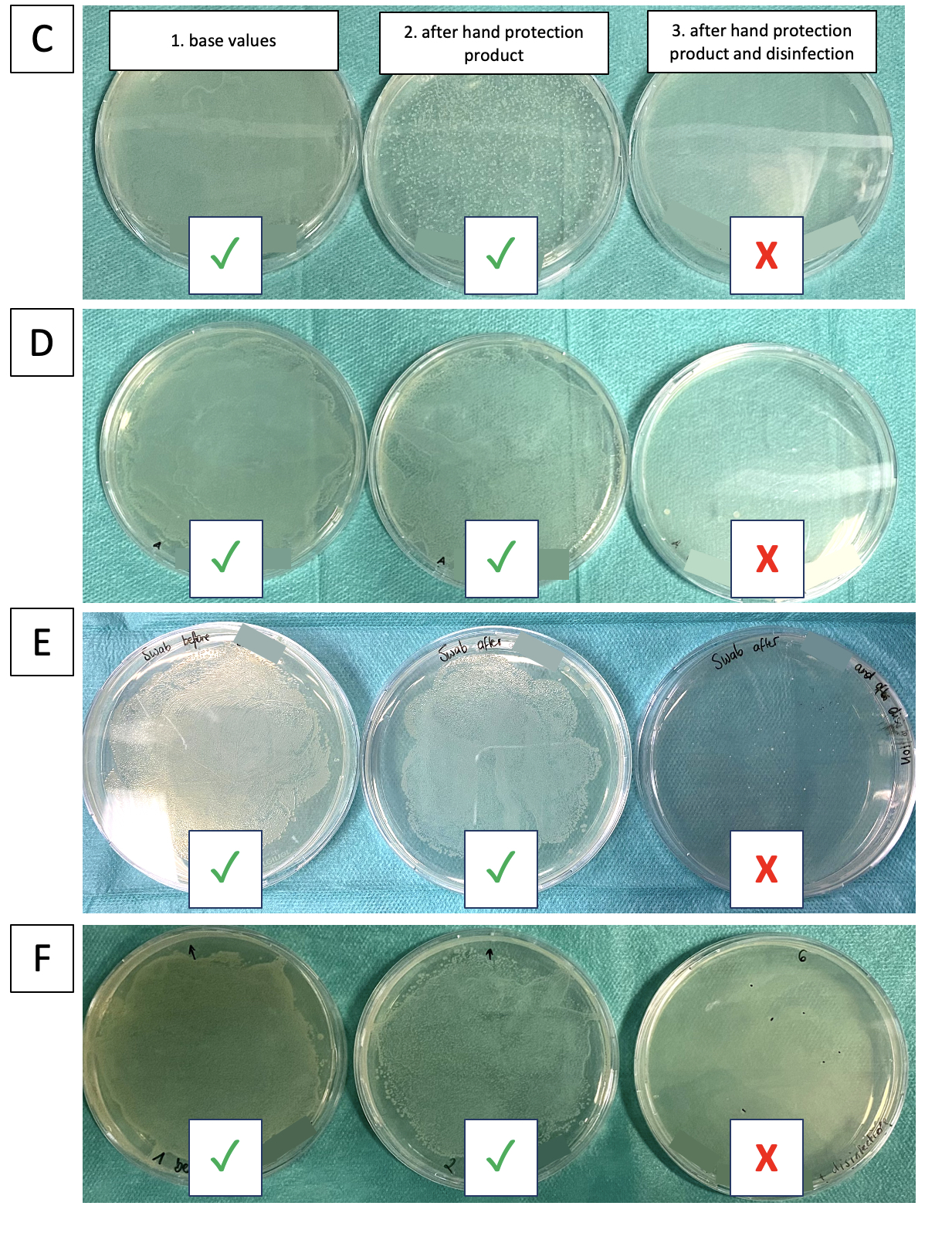

Supplement: Supplementary file 1 [file healthcare-12-00646-s001.zip › supplements/figure S2_all swabs_water insoluble.jpg]

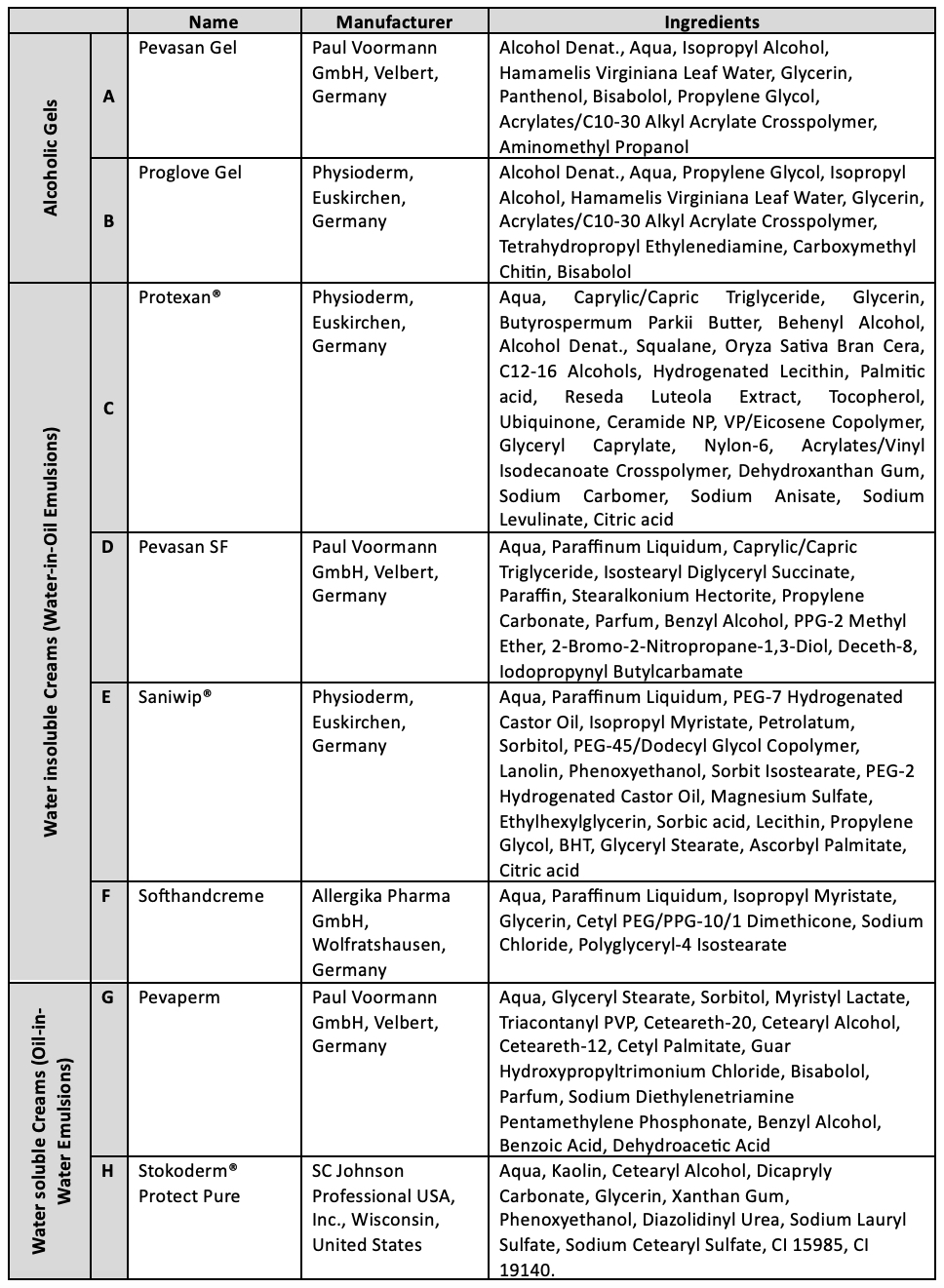

Supplement: Supplementary file 1 [file healthcare-12-00646-s001.zip › supplements/table S1_list of ingredients.jpg]

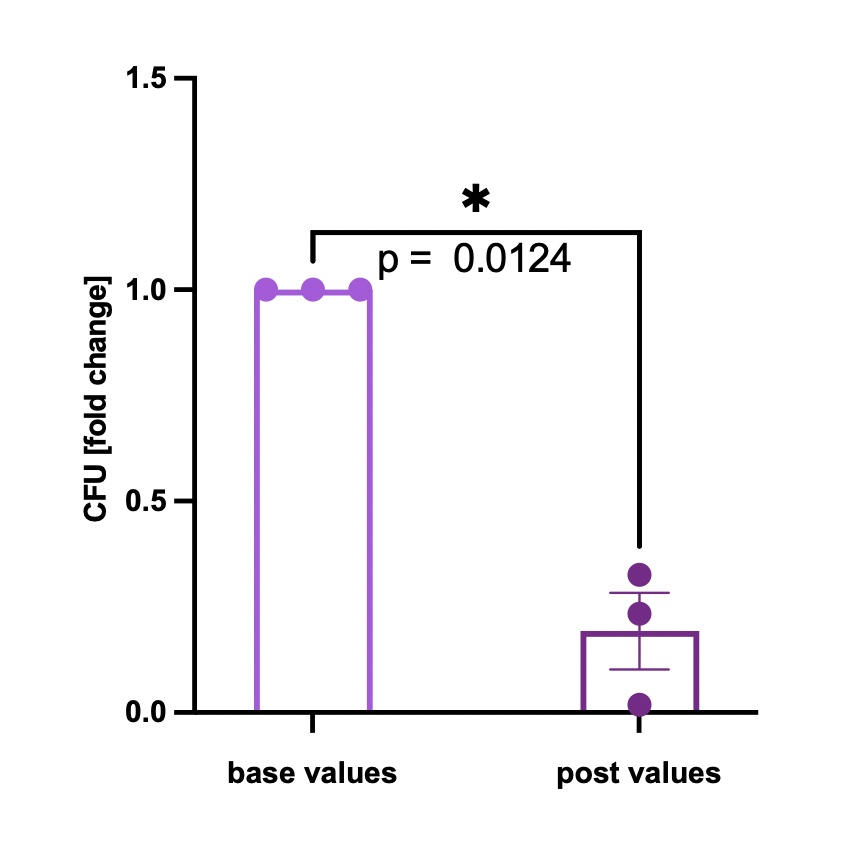

Supplement: Supplementary file 1 [file healthcare-12-00646-s001.zip › supplements/figure S4_necessity for reconatmination.jpg]

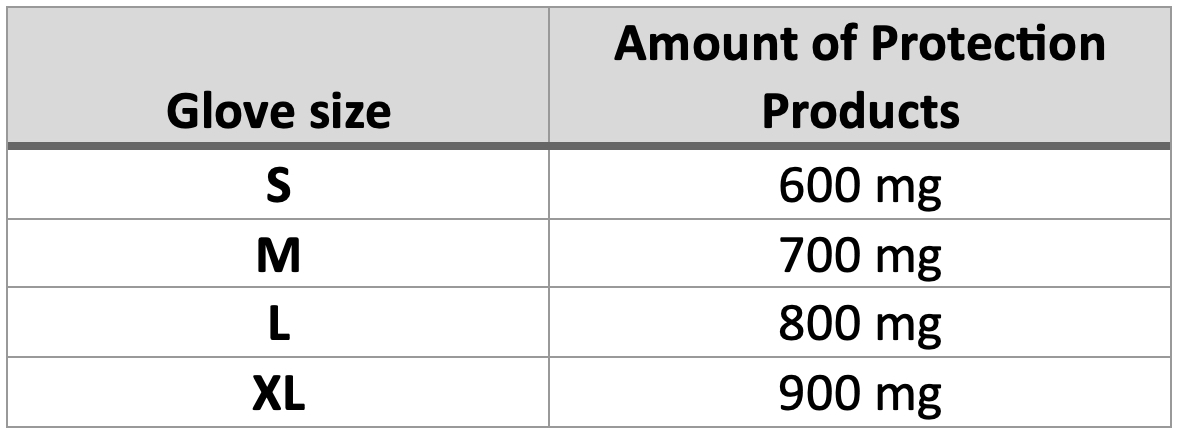

Supplement: Supplementary file 1 [file healthcare-12-00646-s001.zip › supplements/Table S3_amount of PPs.jpg]

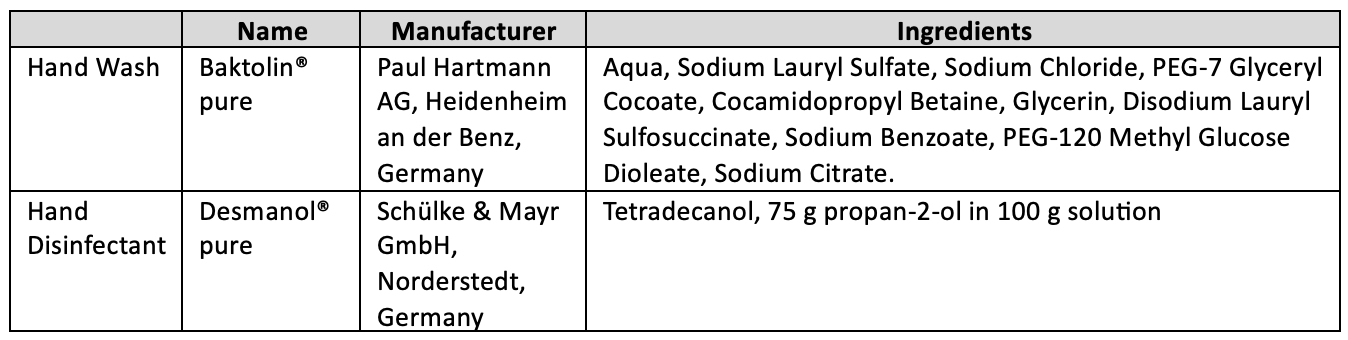

Supplement: Supplementary file 1 [file healthcare-12-00646-s001.zip › supplements/table S2_list of ingredients_soap_disinfectant.jpg]

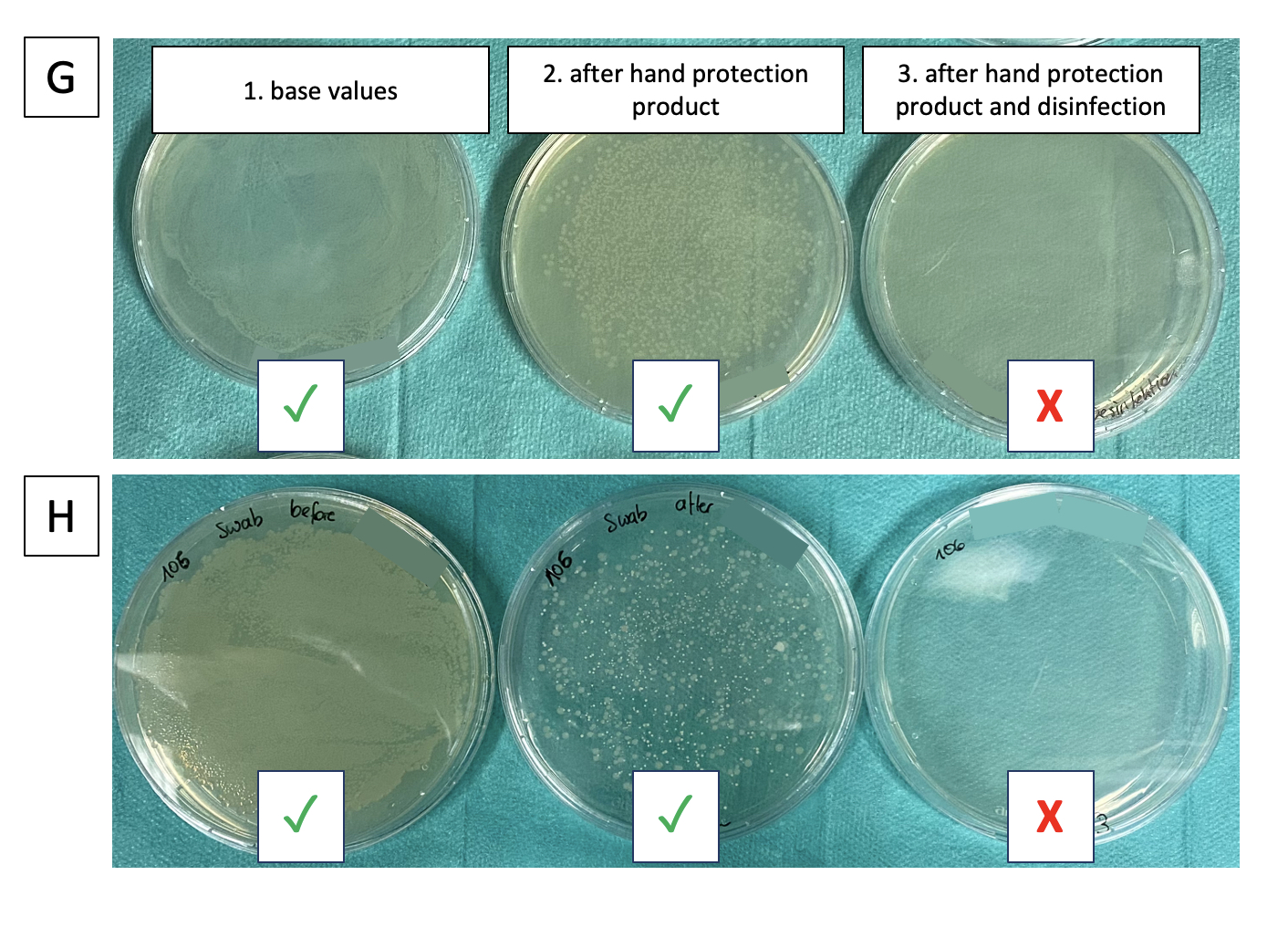

Supplement: Supplementary file 1 [file healthcare-12-00646-s001.zip › supplements/figure S3_all swabs_water soluble.jpg]
